# Enzymatic and chemo-enzymatic strategies to produce highly valuable chiral amines from biomass with ω-transaminases on 2D zeolites

**DOI:** 10.1093/nsr/nwac135

**Published:** 2022-07-29

**Authors:** J Miguel Carceller, Karen S Arias, Maria J Climent, Sara Iborra, Avelino Corma

**Affiliations:** Instituto de Tecnologia Química (UPV-CSIC), Universitat Politècnica de València, Valencia 46022, Spain; Instituto de Tecnologia Química (UPV-CSIC), Universitat Politècnica de València, Valencia 46022, Spain; Instituto de Tecnologia Química (UPV-CSIC), Universitat Politècnica de València, Valencia 46022, Spain; Instituto de Tecnologia Química (UPV-CSIC), Universitat Politècnica de València, Valencia 46022, Spain; Instituto de Tecnologia Química (UPV-CSIC), Universitat Politècnica de València, Valencia 46022, Spain

**Keywords:** amino transaminase, ITQ-2 zeolite, chemo-enzymatic process, biomass, semi-continuous process, Pd/MgO

## Abstract

Amino transaminases (ATAs) have been supported on a 2D ITQ-2 zeolite through electrostatic interactions, resulting in a highly stable active biocatalyst to obtain a variety of valuable chiral amines starting from prochiral ketones derived from biomass. We have extended the biocatalyst applications by designing a chemo-enzymatic process that allows, as the first step, prochiral ketones to be obtained from biomass-derived compounds through an aldol condensation–reduction step using a bifunctional metal/base catalyst. The prochiral ketone is subsequently converted into the chiral amine using the immobilized ATA. We show that it is feasible to couple both steps in a semi-continuous process to produce industrially relevant chiral amines with yields of >95% and ∼100% enantiomer excess.

## Teaser

Valuable chiral amines useful as intermediates in the pharmaceutical industry have been selectively synthesized starting from renewable carbonyl compounds from biomass by coupling a chemical and an enzymatic heterogeneous catalyst through a two-step semicontinuous process.

## INTRODUCTION

Biomass, along with CO_2_, constitutes the only renewable source of carbon compounds as an alternative to fossil fuels to produce chemicals and fuels [1,2]. Particularly from biomass, hundreds of chemical compounds can be obtained through thermal, catalytic and fermentative processes. The specific functional groups existing in these biomass-derived chemicals represent an important advantage for introducing heteroatoms such as N, P, S, etc. Particularly, nitrogen-containing compounds are high-added-value chemicals for the preparation of drugs, agrochemicals, polymers or surfactants in the form of amines, amides, nitriles, heterocyclic compounds, etc. In fact, N-chemicals are present in >80% of the 200 most important products of the pharmaceutical industry and they are usually obtained from petrochemical platforms. For this reason, in recent years, there has been great interest in developing N-chemicals through efficient and sustainable routes from renewable raw materials from biomass [3]. Particularly, optically pure amines are valuable compounds for the pharmaceutical and agrochemical industries [4–13]. However, conventional chemical methods used for the synthesis of chiral amines suffer from several limitations such as low selectivity and high generation of wastes [14]. Thus, during the last two decades, many efforts have been carried out to develop alternative biocatalytic routes. Besides the biocatalytic approaches based on the kinetic resolution of racemic amines (using for example lipases) and the dynamic kinetic resolution (via *in situ* racemization of the unreactive enantiomer) [15–17], diverse types of enzymatic activities have been explored, such as imine reductases, amine dehydrogenases, ammonia lyases, monoamine oxidases and transaminases [18].

**Scheme 1.**
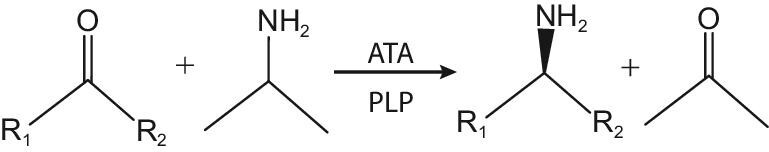
Chiral amines from prochiral ketones using amino transaminases (ATAs).

An interesting group of ω-transaminases are the amino transaminases (ATAs) able to transfer an amino group from a simple amine (donor) to a prochiral ketone (acceptor) (Scheme 1). These enzymes use the cofactor pyridoxal 5^′^-phosphate (PLP) for assisting the reaction that is *in situ* restored at the end of the reaction, and no additional and costly cofactor regeneration is required, as is the case of other enzymes [19–22].

This property, combined with the excellent stereoselectivity shown by that class of enzymes, makes the transaminases potential biocatalysts for the synthesis of chiral amines [23].

Nevertheless, for the large-scale application of transaminases, several problems typically associated with enzymes need to be solved, namely the limited range of substrates that can be used or problems related to enzyme stability and reusability. To overcome these problems, biotechnological strategies to increase the substrate scope of the enzyme, such as rational design or gene mining [24,25], have been developed, while enzyme immobilization over different supports has been the most-used strategy to increase the stability and the reusability of these biocatalysts [22]. However, the moderate operational stability of ATAs upon immobilization has prompted a number of studies on the immobilization of ω-transaminases on different supports directed at improving the overall performance of the biocatalyst. Thus, transaminases have been immobilized over a variety of carriers, such as organic supports as cellulose or chitosan via covalent interaction [26], over macrocellular silica [27], commercial resins as Sepabeads EC-HA [13] via hydrophobic, ionic or covalent interaction. Controlled porosity glass, polymer-hybrid versions (HybCPG) [28] and polydopamine magnetic nanoparticles [29] chelated with Co^+2^ and Ni^+2^, respectively, have been used to immobilize transaminases via electrostatic interactions of metals and histidine residues. Also, bisepoxide-activated polymeric resins [30] and lens-shaped polyvinyl alcohol particles [31] have been recently reported.

An important challenge often associated with ATA catalysed transamination is the unfavorable thermodynamic equilibrium of the reaction. To overcome this drawback, different strategies have been used [32], such as removing the product (or co-product) from the reaction by using different methods. However, a promising alternative is the use of immobilized ATAs in flow systems. Here, as the carbonyl compound is converted into the amine, the latter is removed from the reaction environment, shifting the equilibrium toward amine formation while enzyme inhibition is avoided. However, the effective enzyme immobilization is a crucial factor for successful biotransformations in a flow system.

Recently, we have reported that the pure silica 2D ITQ-2 zeolite is an excellent carrier to support enzymes such as naringinase [33] and alcohol dehydrogenases [34] that have been used efficiently, in both batch and flow mode, in the production of citrus flavonoids as well in the production of chiral alcohols from racemic mixtures through a chemo-enzymatic cascade process respectively. The ITQ-2 zeolite that can be prepared by a dual-templating one-step synthesis or by the delamination of a layered Mobil twenty two (MWW) zeolite precursor [35–38] is a 2D system of 2.5-nm-thick thin sheets. Thus, the ITQ-2 zeolite exhibits high external surface area (∼600 m^2^ g^−1^) where the enzyme can be immobilized with high stability in aqueous media and provides a suitable support for enzyme immobilization through electrostatic or covalent linkages.

Considering that a wide variety of prochiral ketones can be obtained from lignocellulosic biomass through thermal, catalytic or fermentative processes, we present in this work an efficient process to obtain valuable chiral amines from biomass-sourced prochiral ketones using an immobilized ω-transaminase (ATA) on the laminar zeolite ITQ-2 through electrostatic interactions. Moreover, we have designed a chemo-enzymatic cascade process to produce chiral 4-phenyl-2-butanamide derivatives from biomass, such as 4-(4-methoxyphenyl)-2-butanamine and 4-phenyl-2-butanamine. The 4-(4-methoxyphenyl)-2-butanamine is a valuable intermediate in the synthesis of drugs such as Dobutamine, which is widely used for the treatment of heart failure and cardiogenic shock [39], while 4-phenyl-2-butanamine has been recently described as a chiral building block for the synthesis of amides that are modulators of lysophosphatidic acid receptors beneficial for the prevention or treatment of liver, skin and lung diseases [40].

**Scheme 2.**
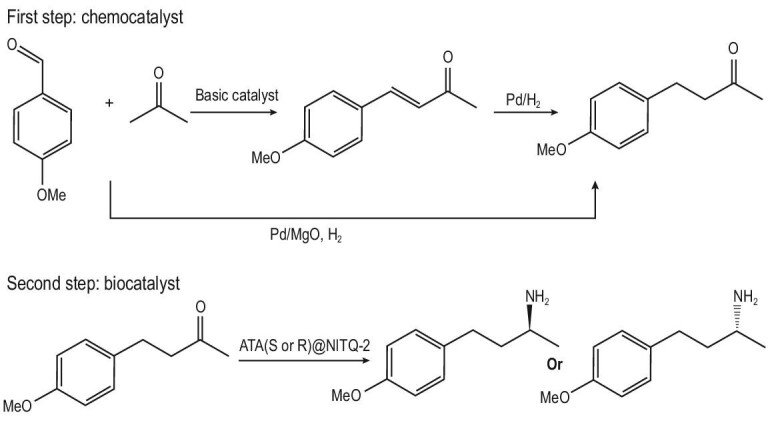
Chemo-enzymatic cascade process.

The chemo-enzymatic route to produce the chiral 4-(4-methoxyphenyl)-2-butanamine involves as the first step the synthesis of the prochiral 4-(4-methoxyphenyl)-2-butanone through a one-pot process performed in the presence of a bifunctional base–metal catalyst formed by Pd nanoparticles supported on MgO. This catalyst is able to perform the aldol condensation between 4-methoxybenzaldehyde (or substituted benzaldehydes that can be sourced from lignin) [41] and acetone (that can be sourced from the acetone-butanol-ethanol (ABE) sugar fermentation process) [42] yielding an α,β-unsaturated ketone that is selectively reduced *in situ* into 4-(4-methoxyphenyl)-2-butanone in the presence of hydrogen [43]. The prochiral ketone is subsequently stereoselectively aminated into 4-(4-methoxyphenyl)-2-butanamine with the enzyme ω-transaminase (ATA) immobilized on a 2D zeolite (ITQ-2) using isopropylamine as the amino group donor (Scheme 2).

## RESULTS AND DISCUSSION

### Characterization of the support and immobilization of ATAs

Transaminases have been immobilized over different supports using different strategies; however, a common methodology is through electrostatic interactions between supports bearing amino groups and the carboxylic acids of the enzyme [13]. Therefore, the surface of the zeolite ITQ-2 was functionalized with amino groups, using (3-aminopropyl)triethoxysilane (3-APS) as described in the experimental section. The resulting material labeled as NITQ-2 contained a density of amino groups of 1.26 mmol g^−1^ as determined by elemental analysis (see Supplementary Table S1).

Both zeolites (ITQ-2 and NITQ-2) were characterized using Fourier transform infrared spectroscopy (FTIR) analysis (Supplementary Fig. S1) As can be observed, the stretching vibration band associated with silanol groups (Si–OH) appears in the ITQ-2 sample at 3735 cm^–1^ (Supplementary Fig. S1a). However, when the surface of the zeolite ITQ-2 is chemically modified with (3-aminopropyl)triethoxysilane through covalent bonding with the silanol groups (NITQ-2 sample), the intensity of the stretching vibration band of the later decreases dramatically, while the bands associated with the amino groups appear at 3422 and 1657 cm^–1^, along with the bands at 2930 and 2865 cm^–1^ associated with –CH_2_ stretching vibration bonds of the propyl group (Supplementary Fig. S1b).

The Brunauer–Emmett–Teller surface area of the zeolite ITQ-2 pure silica was calculated to be 592 m^2^ g^−1^ and, after chemical modification with 3-APS, the Brunauer, Emmett and Teller (BET) surface area decreased by ≤375 m^2^ g^−1^; nevertheless, it represents a high surface area support where the enzyme is electrostatically anchored (see Supplementary Table S2).

FESEM images of ITQ-2 and NITQ-2 (Supplementary Fig. S2a and b, respectively) show the characteristic 2D structure of the material able to carry enzymes with huge sizes and, from tansmission electron microscopy (TEM) images, it is possible to observe the crystallinity of the material (Supplementary Fig. S3a and b).

### Properties of immobilized ATA

First, we optimized the enzyme loading by varying the amount of the enzyme on the support as shown in Table 1. The immobilization of the enzyme (ATA(S)) was performed by incubating different amounts of enzyme with NITQ-2 in a phosphate buffer solution at pH 7 for 24 h. The amount of protein immobilized was determined by analysing the amount of free enzyme in the supernatant using the bicinchoninic acid protein test. As can be observed, when the ratio of the enzyme/support (wt/wt) was increased (Entries 1–4), the amount of immobilized enzyme increased from 12.1 (Entry 1) to 40 mg ATA/g NITQ-2 (Entry 4), while the percentage of immobilized enzyme was maintained at 69%–80%. However, when the ratio enzyme/support was further increased (Entry 5), the immobilization level was considerably lower (49%) while the amount of immobilized enzyme per gram of support slightly decreased (38.0 mgATA/gNITQ-2). Then, an optimized enzyme/support ratio was found under the conditions presented in Entry 4. The catalytic activity of the samples with different loadings was tested taking as a model reaction the amination 4-(4-methoxyphenyl)-2-butanone into (S)-4-(4-methoxyphenyl)-2-butanamine. Reactions were performed at 37°C using a molar ratio substrate/cofactor of 15/1. In Supplementary Fig. S4 the results of the activity for the different samples are presented, measured as the yield of amine produced after 60 min of reaction time versus enzyme loading. As can be seen, the activity of the biocatalyst is directly proportional to the amount of enzyme present on the surface of the solid. Therefore, we selected the sample corresponding to Entry 4 (Table 1) labeled as ATA(S)@NITQ-2 with 81% of enzyme immobilization for performing all the subsequent studies. To compare the intrinsic activity of the free and supported enzyme, the activity recovery was calculated as the activity of immobilized enzyme/activity of free enzyme × 100, being 62%.

**Table 1.** Optimization of the enzyme loading over the zeolite NITQ-2.

Entry	ATA(S) (mg)	NITQ2 (g)	Immobilization conditions mgATA/gNITQ-2	Immobilization (%)	Ratio mgATA/g NITQ-2
1	3	0.2	15	81	12.1
2	5	0.2	25	69	17.2
3	4	0.1	40	71	28.4
4	5	0.1	50	80	40.0
5	8	0.1	80	49	38.0

### Catalytic activity as a function of pH and thermal stability

To study the effect of pH on the transamination of 4-(4-methoxyphenyl)-2-butanone, the activity of ATA(S)@NITQ-2 was studied at pH 5, 7 and 9 at 37°C and compared with that of the free enzyme (see Supplementary Fig. S5). As can be observed, the free and supported form of the enzyme showed the maximum activity at pH = 7, while acidic pH (5) or basic pH (9) was detrimental for the enzyme activity, particularly acidic media.

The thermal stability of ATA(S)@NITQ-2 was determined by exposing the supported enzyme to different temperatures: 37, 60 and 80°C for 1 h at pH 7. After that, the substrate 4-(4-methoxyphenyl)-2-butanone and the cofactor (PLP) was added and the residual catalytic activity was determined under the conditions described in the Supplementary data. In Supplementary Fig. S6, the residual relative activities of the free and immobilized enzyme after the thermal treatment are presented. As can be seen, at 37°C, the free enzyme performs faster than the immobilized form, giving ∼100% activity and 60%, respectively. After treatment at increasing temperatures, both the immobilized and free enzyme undergo a strong loss of activity. In fact, the free enzyme lost its activity completely at 60°C, while the immobilized enzyme retained a residual activity. It has been described that enzyme immobilization can restrict unfolding of the enzyme and therefore the thermal stability could be somewhat improved [44]. However, the results show that the optimum temperature of the biocatalytic process should not surpass 37°C in order to maintain high enzymatic activity.

### Scope of the transamination with ATA(S)@NITQ-2

As commented on above, there are a variety of prochiral ketones than can be obtained from biomass and they can serve as starting reactants to produce chiral amines useful in the fine chemical industry. Therefore, we have selected a variety of prochiral ketones with phenylethanone and 1-(4-methoxyphenyl)propanone structures that can be obtained from p-coumaril alcohol derivatives from lignin [41] and which chiral amines derivatives (1-phenylethylamine, 1-(4-methoxyphenyl)-2-propanamine) are important chiral inductors and building blocks for the synthesis of drugs such as Brivaracetam, Fenoterol and Formoterol, used as anticonvulsant, anti-asthmatic and bronchodilator agents, respectively [45,46]. In addition, several linear (C_7_–C_9_) 2-methylketones that can be obtained through biomass fermentation processes [47] or by catalytic cross ketonization of carboxylic acids from biomass [48] can be prochiral substrates for the production of aliphatic amines that are important intermediates of pharmaceuticals and other biologically active compounds [23]. Moreover, the ketoester methyl levulinate was also selected as a prochiral precursor. The scope of the reaction was studied using the enzymatic derivative ATA(S)@NITQ-2 under the optimal reaction conditions in batch mode and the results are presented in Table 2. As can be observed, excellent yields of the corresponding chiral amines (80%–98%) could be obtained in short reaction times, while the enantiomer excess (ee) was 99% in all cases, showing the outstanding performance of ATA(S)@NITQ-2 biocatalyst on a variety of biomass-derived substrates. When the enantiocomplementary enzyme (ATA(R)@NITQ-2) (Entry 1) was used for the amination of 4-phenyl-2-butanone, the yield and enantioselectivity to the (R)-4-phenylbutan-2-amine were also very high, although a longer reaction time was required. The amination of methyl levulinate (Entry 8, Table 2) afforded (S)-5-methylpyrrolidin-2-one after spontaneous cyclization of the intermediate product (S)-methyl 4-aminopentanoate.

**Table 2.** Transamination of different ketones using ATA@NITQ-2 as the biocatalyst.

Entry	Reactive	Product	Time (h)	Yield (%)/ee(%)
1			112	(S) 96/99(R) 95/99
	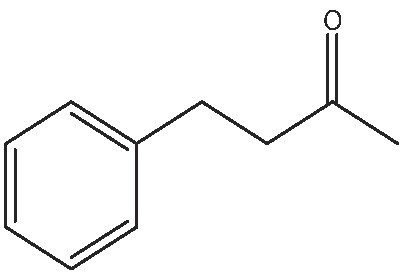	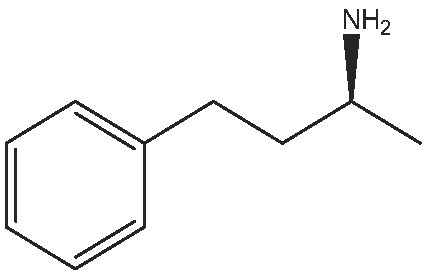		
2			1	(S) 98/99
	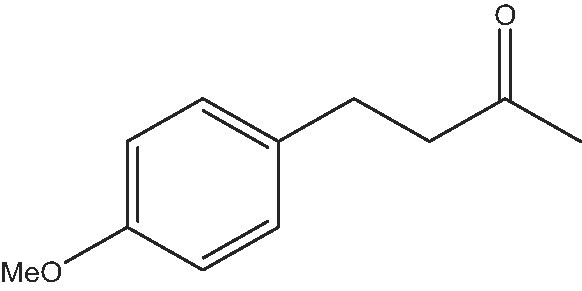	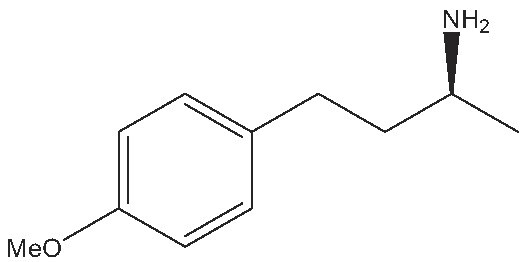		
3			1	(S) 86/99
	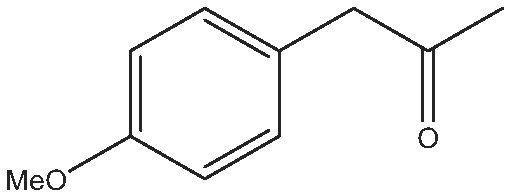	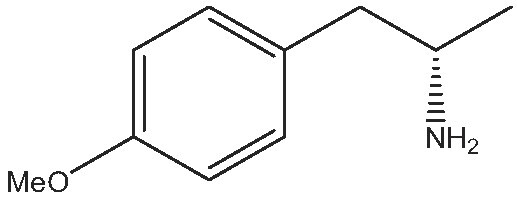		
4			2	(S) 80/99
	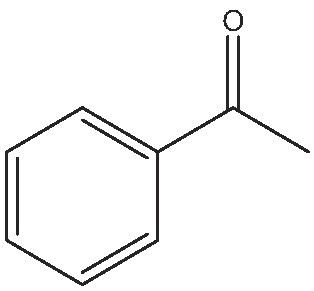	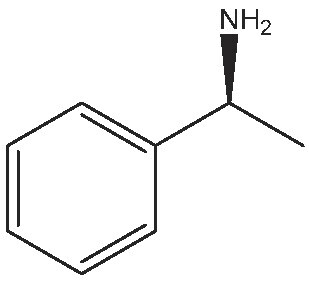		
5			2	(S) 86/99
	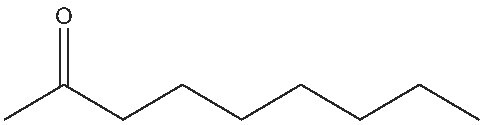	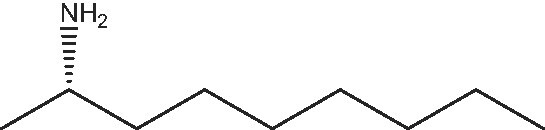		
6			2	(S) 84/99
	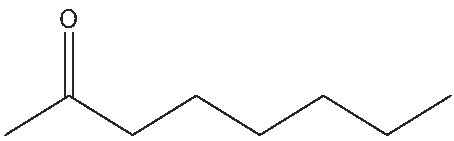	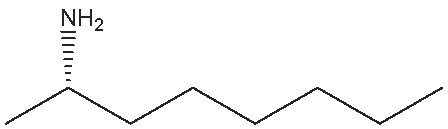		
7			2	(S) 88/99
	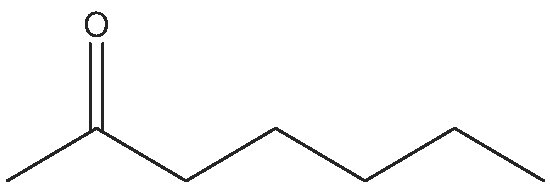	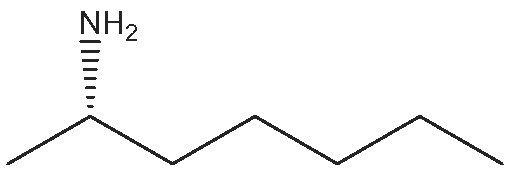		
8			24	(S) 90/99
	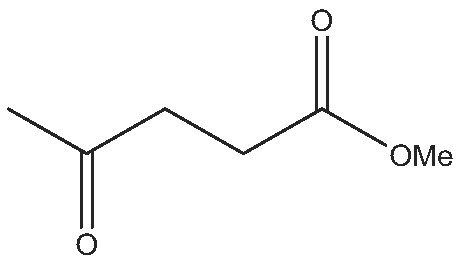	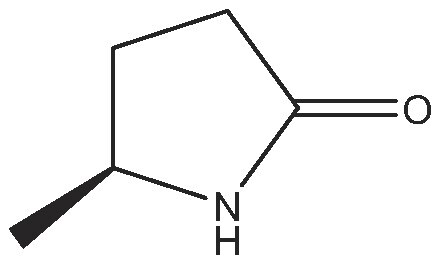		

Reaction conditions: ATA@NITQ-2 (104 mg), ketone (15 mM), the cofactor (PLP) (1 mM) in 2 mL of solvent (isopropylamine (pH 7, 2 M)/phosphate buffer solution, pH 7, 100 mM) (50/50, v/v) and DMSO (10%) as co-solvent, at 37°C while stirring at 1400 r/min.

### Enzymatic amination of the prochiral ketone in a continuous fixed-bed reactor

**Figure 1.**
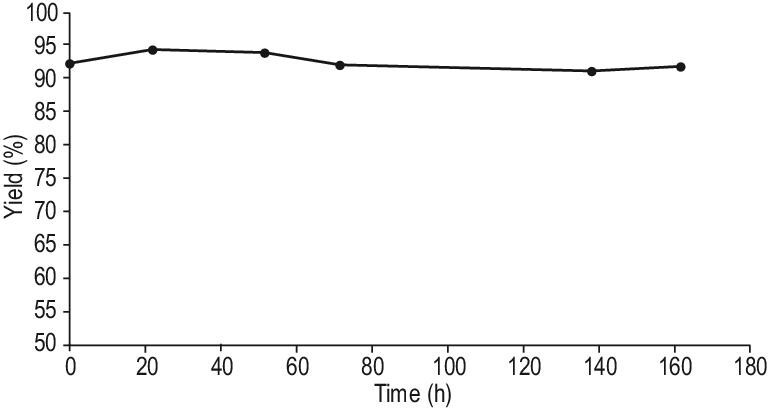
Results of the transamination of 4-(4-methoxyphenyl)-2-butanone to (S)-4-(4-methoxyphenyl)-2-butanamine in a continuous flow reactor using ATA(S)@NITQ-2 as the biocatalyst. Reaction conditions: ATA(S)@NITQ-2 (208 mg), 4-(4-methoxyphenyl)-2-butanone (15 mmol L^−1^), PLP (1 mmol L^−1^), solvent: isopropylamine/phosphate buffer pH 7, 100 mM (50/50 v/v) and DMSO (10%) as co-solvent, flow 0.25 mL h^–1^ at 37°C.

The amination of the prochiral ketone 4-(4-methoxyphenyl)-2-butanone was performed in a flow reactor system. Initially, the biocatalyst ATA(S)@NITQ-2 (208.0 mg) was diluted with silicon carbide CSi (1.8 g) and packed in a stainless-steel reactor. The reactor was fed through a peristaltic pump with a solution 4-(4-methoxyphenyl)-2-butanone and the cofactor (in a substrate/cofactor molar ratio of 15) using a mixture of isopropylamine/phosphate buffer pH 7, 100 mM (50/50 v/v) as a solvent and 10% DMSO as co-solvent. The temperature was fixed at 37°C and the contact time was evaluated keeping constant the amount of 4-(4-methoxyphenyl)-2-butanone and increasing the flow (0.25–3 mL h^–1^). As we can observe (Supplementary Fig. S7 and Supplementary Table S3), when increasing the contact time from 1 to 7 h, the conversion increased from 50% to 71%, respectively (∼100% selectivity to amine); a further increase in the contact time to 13 h led to 94% conversion maintaining the selectivity to the optically pure S-4-(4-methoxyphenyl)2-butanamine at ∼100%. Then, we selected a contact time of 13 h (that corresponds to a weight hour space velocity (WHSV) of 0.076 h^−1^) to check the performance of the enzymatic derivative. As can be seen in Fig. 1, the conversion of 4-(4-methoxyphenyl)-2-butan-one (93%) could be maintained during 160 h of operation. In fact, the reactor was kept under operation for 20 days, maintaining its activity. Similar data were obtained using the ATA(R)@NITQ-2, obtaining in this case 4-methoxyphenyl-(R)-2-butanamine with ∼100% selectivity (data not show). To study the possible catalyst deactivation, the reaction was performed using a contact time of 1 h, which afforded a conversion of ∼50% and, as can be seen in Supplementary Fig. S8, under these reaction conditions, the biocatalyst activity was maintained during the period studied. Moreover, the presence of leached enzyme in the reaction crude (using the bicinchoninic acid protein test) was not detected during the time-on-stream, confirming the high stability of the biocatalyst.

The continuous amination process using the ATA(S)@NITQ-2 biocatalyst was also extended to 4-phenyl-2-butanone, obtaining excellent yield and selectivity to the corresponding chiral amine (see Supplementary Figs S9 and S10, respectively). These results reveal the high stability of the biocatalyst that in this experiment was able to produce ∼40 g of chiral amine per gram of enzyme. In Supplementary Table S4 the productivity and operational stability of different supported transaminases are compared for the amination of different carbonyl compounds previously reported in the literature. As can be seen, the biocatalyst developed in this work is among the most active, representing an interesting alternative to produce chiral amines from biomass-derived prochiral ketones.

### Chiral amines trough a chemo-enzymatic process

We have shown that ATA@NITQ-2 is a stable biocatalyst able to perform the stereoselective amination of prochiral ketones into the corresponding enantiopure amines. Following the objective stated above, we studied the possibility of coupling the synthesis of a prochiral ketone with the amination process into a chiral amine. Owing to the pharmacological interest of chiral 4-methoxyphenylbutanamines, we performed the synthesis of the prochiral ketone precursor (4-methoxyphenyl-2-butanone) as a reaction model through the aldol condensation of 4-methoxybenzaldehyde and acetone (both available molecules from biomass), followed by hydrogenation of the C=C bond in one pot (see Scheme 2) using a bifunctional catalyst (Pd/MgO) bearing basic sites (able to perform the aldol condensation) and metal sites (able to catalyse the hydrogenation of the C=C bond). Previously, we had shown that this process is a highly sustainable route to producing the target ketone as the calculated E factor is 0.01, water being the main waste [49].

As can be observed in Supplementary Fig. S11, the catalyst performs the one-pot reaction with high selectivity, achieving total conversion of the 4-methoxybenzaldehyde in 2 h with 93% selectivity to 4-(4-methoxyphenyl)-2-butanone. Moreover, the reusability of the Pd/MgO catalyst was studied by performing the reaction trough three consecutive cycles. As can be observed, the activity of the catalyst was maintained in consecutive runs, demonstrating the high stability of this catalytic system (see Supplementary Fig. S11).

Additionally, the one-pot reaction was tested with another aldehyde (benzaldehyde) under the same reaction conditions giving similar results of benzaldehyde conversion (99%) and 94% selectivity to 4-phenyl-2-butanone.

**Figure 2.**
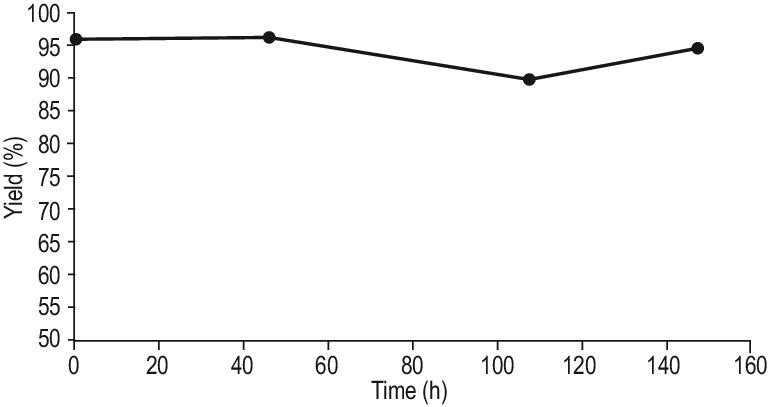
Results of the combined semi-continuous chemo-enzymatic process to produce (S)-4-(4-methoxyphenyl)-2-butananime from 4-methoxybenzaldehyde and acetone. Reaction conditions: ATA(S)@NITQ-2 (208 mg), 4-(4-methoxyphenyl)-2-butanone (15 mmol L^−1^), PLP (1 mmol L^−1^), solvent: isopropylamine/phosphate buffer pH 7, 100 mM (50/50 v/v) and DMSO (10%) as co-solvent, flow 0.25 mL h^–1^ at 37°C.

**Scheme 3.**
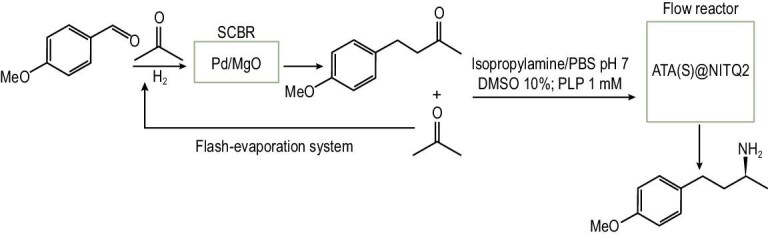
Semi-continuous chemo-enzymatic process.

To explore the efficacy of our proposal for the synthesis of enantiopure primary amines, both steps were coupled in a chemo-enzymatic process carried out in a semi-continuous batch reactor (SCBR) for the aldol-reduction step (first step) combined with an fixed-bed continuous reactor for the transamination reaction step. Thus, after the first step in the SCBR reactor, the excess of acetone was eliminated from the reaction mixture and the 4-(4-methoxyphenyl)-2-butanone (1.44 mmol) was diluted with 95 mL of solvent (isopropylamine/phosphate buffer pH 7, 100 mM (50/50 v/v) and DMSO (10%)) and the cofactor PLP was added (1 mM). Then, the fixed-bed reactor where the ATA(S)@NITQ-2 was placed was fed with this mixture. The continuous reactor was maintained at 37°C and the contact time was 13 h (that corresponds to a WHSV of 0.076 h^−^^1^). Under the optimized conditions, the biocatalytic amination of 4-(4-methoxyphenyl)-2-butanone takes place giving (S)-4-(4-methoxyphenyl)-2-butanamine, in 96% conversion and 99% ee. In good agreement with the results presented in Fig. 1, the catalytic activity was maintained for at least 150 h without observing any deactivation (Fig. 2). The results presented above show that it is feasible to couple both steps (one-pot aldol-reduction reaction with the stereoselective amination of the prochiral ketone intermediate) in a semi-continuous process for obtaining pharmaceutical products starting from prochiral biomass-derived ketones. A schematic representation of the process is presented in Scheme 3. In this system, after condensation between the aldehyde and acetone, the acetone excess can be evaporated using a flash-evaporation system and recollected to be used again in the condensation step. In the second step, the on stream of the methyl ketone formed (after adjusting their concentration and pH) along with the required additives (amino-donor, cofactor and co-solvent) is introduced at the top of the catalytic bed where the supported transaminase is placed.

Interesting aspects of the chemo-enzymatic process are the low E factor of the first step (aldol condensation coupled with reduction) and the possibility of recovery and reuse in the first step of the by-product (acetone) produced in the enzymatic reaction, leading to a high atom-economy process.

## CONCLUSION

We have shown that ATAs electrostatically supported on a 2D ITQ-2 zeolite resulted in a highly stable active biocatalyst to obtain a variety of valuable chiral amines starting from prochiral ketones sourced from biomass. Moreover, we have expanded the biocatalyst applications by designing a new fully sustainable catalytic process by means of chemo-enzymatic systems with a very high atom economy, which allows process intensification. More specifically, we have introduced a catalytic process that involves a bifunctional metal/base solid catalyst and the enzyme transaminases supported on a 2D zeolite to prepare chiral amines with yields of >95% and ee of ∼100% from biomass-derived compounds that can take place in a cascade mode.

## METHODS

### Enzymatic reactions in batch reactor

Reactions were performed by incubating the immobilized enzyme ATA(S)@NITQ-2 with the ketone (4-(4-methoxyphenyl)-2-butanone) (15 mM), the cofactor (PLP) 1 (mM) in 2 mL of solvent (isopropylamine (pH 7, 2 M)/phosphate buffer solution, pH 7, 100 mM) (50/50 v/v) and DMSO (10%) as co-solvent, at 37°C while stirring at 1400 r/min. After the reaction, the biocatalyst was separated from the mixture by centrifugation at 6000 r/min for 5 minutes, washed with phosphate buffer (pH 7) and stored at 4°C until use. The reaction mixture was supplemented with NaOH 8 M and then extracted using methyl tert-butyl ether and dried with anhydrous MgSO_4_. The organic phase was analysed on a Varian 3900 gas chromatograph equipped with a capillary column HP-5 (30 m × 0.25 mm × 0.25 μm) and flame ionization detector. Dodecane was used as the external standard and the molar balance was in all cases >96%.

### Transamination reactions of prochiral ketones in a fixed-bed reactor

For experiments in the flow reactor, a dry sample of ATA(S)@NITQ-2 (208 mg) was diluted with silicon carbide (CSi ≥ 0.25 μm, 1.8 g) and packed in a stainless-steel reactor (diameter 1 mm, height 35 mm). The catalyst dilution with the inert solid particle, having the same size as the catalytic particles, reduces the local hot-spots and improves the temperature distribution along the catalytic bed. The dilution of the catalyst was performed following the literature [50] where it is recommended that the volume of the bed (catalyst plus inert) has to fit in a range depending on the diameter and length of the reactor used. The temperature was fixed at 37ºC for all experiments and the reactor was coupled with a peristaltic pump for feeding. To evaluate the contact time, the flow rate was increased from 0.25 to 3 mL h^−1^ while the temperature and the concentration of 4-(4-methoxyphenyl)butan-2-one dissolved in the feeding (isopropylamine (pH 7, 2 M)/phosphate buffer solution, pH 7, 100 mM) (50/50 v/v), DMSO (10%) as co-solvent, 1 mM PLP were keep constant. The contact time (h) has been calculated as the inverse of space velocity WHSV (h^−1^), which is defined as: WHSV (h^−1^) = limiting reactant (g h^−1^)/gram of catalyst.

### Chemo-enzymatic process

The coupling of both reactors was carried out as follows: the one-pot reaction procedure to obtain 4-(4-methoxyphenyl)-2-butanone was performed in a SCBR; 4-methoxybenzaldehyde (1.94 mmol), acetone hplc grade (70 mmol) and dodecane as internal standard were charged in a 13-mL stainless-steel autoclave reactor with a Teflon vessel containing a magnetic stirring bar. When the desired temperature (100ºC) was reached, the catalyst sample Pd/MgO was added 5 wt% and hydrogen at a constant hydrogen pressure of 5 bar was applied for 2 h while stirring at 750 r/min. Then the acetone solution was concentrated under reduced pressure on a Büchi rotary evaporator. After that, the 4-(4-methoxyphenyl)-2-butanone (1.44 mmol; 94% yield) obtained from the first reactor SCBR was diluted with a mixture of (isopropylamine (pH 7, 2 M)/phosphate buffer solution, pH 7, 100 mM) (50/50 v/v), DMSO (10%) as co-solvent until a volume of 95 mL. To this solution of 4-(4-methoxyphenyl)-2-butanone (15 mM), the cofactor (PLP) (1 mM) was added and used for feeding the second reactor (ADH(S)@NITQ-2) at a flow of 0.25 mL h^−1^. The temperature was fixed at 37ºC for all experiments and the reactor was coupled with a peristaltic pump for feeding.

## Supplementary Material

nwac135_Supplemental_File
