# Supplementary material for: Enzymatic and chemo-enzymatic strategies to produce highly valuable chiral amines from biomass with ω-transaminases on 2D zeolites
Source: Natl Sci Rev. 2022 Jul 29;9(9):nwac135. doi: 10.1093/nsr/nwac135 (PMC9479500; doi:10.1093/nsr/nwac135)
Supplement: nwac135_Supplemental_File [file nwac135_supplemental_file.pdf]

Supplementary Data

Enzymatic and chemo-enzymatic strategies to produce highly valuable chiral amines from biomass with ω-transaminases on 2D zeolites

J. Miguel Carceller, Karen S. Arias, Maria J. Climent, Sara Iborra*, Avelino Corma*

Instituto de Tecnologia Química (UPV-CSIC).
Universitat Politècnica de València
Avda dels Tarongers s/n, 46022, Valencia (Spain)

Materials

Prelog Transaminases (ATA47) (S) and antiPrelog (ATA87) (R) were purchased from Codexis, 4-phenylbutan-2-one, acetophenone, 4-(4-methoxyphenyl)butan-2-one, 1-(4-methoxypenylpropa-2-one, 2-nonanone, 2-octanone, 2-heptanone, methyl levulinate and the cofactor pyridoxal 5'-phosphate (PLP) were purchased from Sigma Aldrich. The pure chiral compounds (R and S) 4-phenyl-2-butanamine, 1-phenylethanamine, 4-(4-methoxyphenyl)-2-butanamine, 4-methoxypenyl-2-propanamine, 2-nonamine 2-octanamine, 2-heptanamine and 5-methylpyrrolidin-2-one were purchased from Enamine Store.

Analytical methods

Infrared analysis of the support was performed with a IR Vertex Burker DTGS (Detector), and a conventional quartz infrared cell Quartz KR55 windows connected to a vacuum dosing system. The samples were pressed into self-supporting pellets and treated under vacuum (10^{-4} to 10^{-5} Pa) at 400 °C for 24 hours.

The specific surface areas of the supports were calculated by the Brunauer–Emmett–Teller (BET) method by means of nitrogen adsorption at −196 °C, using an ASAP 2420 (V2.09 J). Elemental analysis (Table S1) was performed in a Euro EA3000 Elemental Analyzer (EuroVector), using sulphanilamide as the reference. The metal content in the samples were analysed by inductively coupled plasma atomic emission spectroscopy (ICP-AES) using a Varian 715-ES. Samples for electron microscopy studies were prepared by dropping the suspension of the powder sample using CH_2Cl_2 as the solvent directly onto holeycarbon coated copper grids. All the measurements of transmission electronic microscopy (TEM) were performed in a JEOL 2100F microscope operating at 200 kV while Field emission scanning electron microscopy (FESEM) measurement was performed with a ZEISS Ultra 55 FESEM. The solid powder sample was adsorbed on conductive carbon tape. Powder X-ray diffraction (XRD) were performed using a PAnalytical CubiX diffractometer using Cu Kα radiation and a multisampling handler.

The identification of the reaction products was carried out by using GC-MS on an Agilent 5973 Network Mass selective Detector equipped with a capillary column HP5-MS Ultrainert (30 m × 0.25 mm × 0.25 μm) and a FID detector. The GC-MS spectra of the amines produced were compared with that of the pure commercial amines. Enantiomeric excess of the amine was determined on a Varian 3900 gas chromatograph equipped with a Supelco β-Dex225 (30 m × 0.25 mm × 0.25 μm) and FID detector, and previous derivatization of the amines with acetic anhydride or trifluoroacetic anhydride following the method described in the literature.[1] Typically, the amine (1 mmol), and DMAP (4-dimethylaminopyridine) (0.1 mmol) was dissolved in ethyl acetate (20 mL) and then anhydride acetic (20 mmol) was added and the mixture was stirred at 40 °C for 3 h. After this time, distilled water was added to hydrolyse the excess of acetic acid anhydride. After drying the organic phase (Na_2SO_4) the ee of the derivatized compound was measured by

GC on a chiral phase Supelco β-Dex column. The identity of the chiral products was verified by comparing the retention times with those of the pure chiral commercial amines, under the same derivatization conditions.

Catalyst preparation and characterization

Synthesis of the pure silica MWW (MCM-22) zeolite was performed following the literature.[2] The procedure is as follows: 0.95 g of NaCl are added in 50.70 g of a solution of 0.42 M N,N,N-trimethyl-1-adamantanamonium hydroxide, previously diluted in 21.33 g of water. Then, hexamethyleneimine (2.62 g) are added to this solution, followed by 4.88 g of silica (Aerosil 200, Degussa) under continuous stirring. The reaction mixture is heated in a Teflon lined stainless steel autoclave at 150 °C rotated at 60 rpm for 5 days. After filtering, the white solid obtained is washed until pH was less than 9, and finally dried at 100 °C. The synthesis of the pure silica ITQ-2 zeolite was carried out following the literature.[2] Initially, 5 g of the pure silica MCM-22 zeolite were added in 20 g of water. Then, 100 g of an aqueous solution of hexadecyltrimethylammonium hydroxide (25% by weight, 50% exchange Br/OH), and 30 g of an aqueous solution of tetrapropylammonium (40% by weight, 30% exchange Br/OH) were added. The resulting mixture (pH 12.5) was heated to 55 °C and stirred vigorously for 16 h to facilitate swelling between zeolitic sheets. At this point, the suspension was treated in an ultrasonic bath (50 W, 50 Hz) for 1 h to disperse the zeolitic sheets. By adding HCl (6 M), the pH decreased to about 3, to facilitate flocculation of the delaminated solid, which is recovered by centrifugation. The solid was washed with distilled water, dried at 60 °C for 12 h, and calcined at 540 °C, first for 3 h in a N_2 atmosphere, and then for 6 h in air.

To functionalise the pure silica zeolite ITQ-2 pure silica with amino groups, the zeolite (500 mg) was activated at 200 °C under vacuum for 2 hours. After cooling at room

temperature, anhydrous toluene (50 mL) and (3-aminopropyl) triethoxysilane (240 μL) were added to the solid and the mixture was refluxed for 24 h at 120 °C. Then, the solid was filtered under vacuum and washed with toluene and n-hexane, obtaining the material that was labelled as NITQ-2, functionalized with amino groups.[3,4]

Magnesium oxide (MgO) with a surface area of 360 m^2/g was purchased from NanoScale Materials. Pd/MgO catalyst was obtained by contacting the MgO sample with an anhydrous toluene solution with palladium acetylacetonate [$Pd(acac)_2$] for 12 h. Then, toluene was evaporated at reduced pressure and the solid was dried overnight at 80 °C in vacuum and then calcined in nitrogen flow at 550 °C (ramp rate, 5 °C /min) for 3.5 h. Samples of Pd/MgO were activated before reaction by heating the solid at 450 °C under air atmosphere for 5 h and then for 5 h under nitrogen. Metal reduction was performed by heating the solid at 450 °C in a flow of H_2/N_2 (90/10) for 2 h. The catalysts thus prepared, had a Pd content of 1.8% (according to ICP analysis) and was characterized by XRD, TEM, and SEM (Figure S10 and S11). After reaction, Pd/MgO catalyst was regenerated by calcination of the solid at 450 ºC under air atmosphere for 5 h and then under nitrogen for 5 h. Reduction of the Pd was performed by heating the solid at 450 ºC in a flow of H_2/N_2 (90/10) for 2 h.

Immobilization of Transaminase on ITQ-2 zeolite

The immobilization of the enzymes ATA(S) and ATA(R) was performed as follows: 5 mg of ATA (R or S) was dissolved in 8 mL of phosphate buffer solution 100 mM (PBS) pH = 7, and subsequently, 100 mg of NITQ-2 was added and left in a closed flask under gentle agitation for 24 hours. After this time, the amount of protein immobilized was determined by the bicinchoninic acid protein test. To do that, 2 mL of the bicinchoninic acid test solution was added to 0.1 mL of the sample and after incubation of the sample

at 37 °C for 30 minutes, the absorbance at λ 562 nm was measured.[5] Finally, the biocatalyst was centrifuged washed thoroughly and stored at 4 °C.

Catalytic experiments

Synthesis of 4-(4-methoxyphenyl)-2-butanone through one-pot reaction using Pd/MgO catalyst.

In a typical procedure, 4-methoxybenzaldehyde (1.94 mmol), acetone (70 mmol), and dodecane as internal standard were charged in a 13 mL stainless steel autoclave reactor with a Teflon vessel containing a magnetic stirring bar. When the desired temperature (100 ºC) was reached, 5 wt % of the catalyst Pd/MgO was added and hydrogen at a constant hydrogen pressure of 5 bar was applied for 2 h while stirring at 750 rpm. Finally, the catalyst was removed by centrifugation at 6000 rpm for 5 min. The reaction was followed by taking samples at regular periods that were analysed by using a Varian 3900 gas chromatograph equipped with a capillary column HP-5 (30 m × 0.25 mm × 0.25 µm) and FID detector. The molar balance in all cases was >95%. The identification of the products was carried out by using a GC-MS on an Agilent 5973 Network Mass selective detector equipped with a capillary column HP5-MS Ultrainert (30 m × 0.25 mm × 0.25 µm) and FID detector. The retention time and GC-MS spectra of the ketones produced were compared with that of pure commercial ketones.

The effect of pH on the catalytic activity of the ATA(S) in free and immobilized forms was determined by incubating the enzyme for 1 hour in the presence of the substrate (4-(4-methoxyphenyl)-2-butanone) under the conditions described in experimental section, in a pH range from 5 to 9.

The thermal stability of the enzyme was determined by heating for 1 hour the free and the immobilized ATA(S) at temperatures between 37 and 80 °C. Subsequently the cofactor

and the substrate (4-(4-methoxyphenyl)-2-butanone) were added and the enzyme activity was determined.

Activity recovery of the biocatalyst was determined according to the literature.[6] First the activity of 5 mg of free ATA(S) for the amination of 4-(4-methoxyphenyl)-2-butanone (15 mM), was determined as described above. The activity was expressed as the rate of (S)-4-(4-methoxyphenyl)-2-butanamine produced (μmol min−1). Then, 5 mg of ATA(S) were contacted with 100 mg of NITQ-2 for 24 hours as described above. After that, the solid was recovered by centrifugation and the activity of the total amount of immobilized enzyme was determined under the same conditions as for the free enzyme. Activity recovery is expressed as a percentage: (activity of immobilized enzyme/activity of free enzyme) × 100.

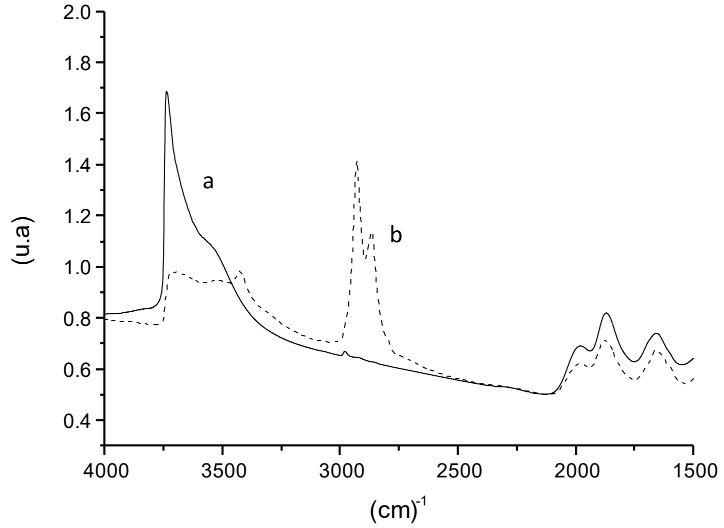

Figure S1. FTIR analysis of ITQ-2 pure silica (a); NITQ-2 (b)

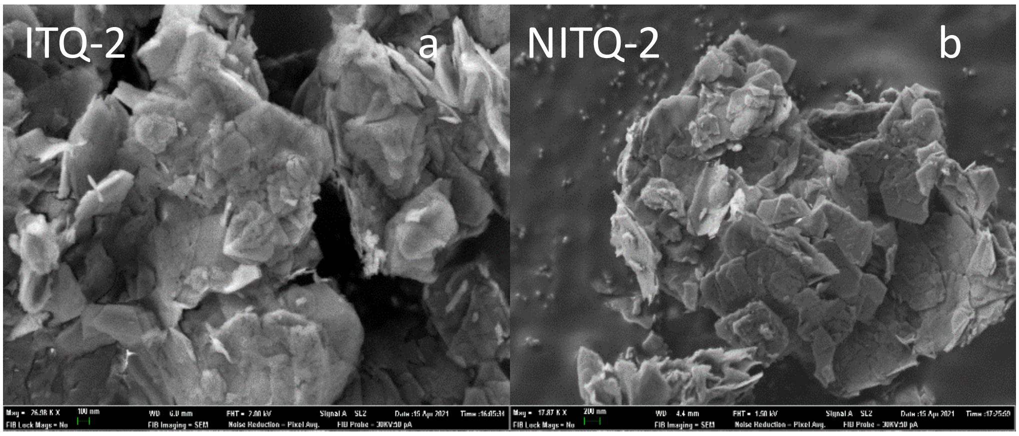

Figure S2. SEM images of ITQ-2 (a) and NITQ-2 (b)

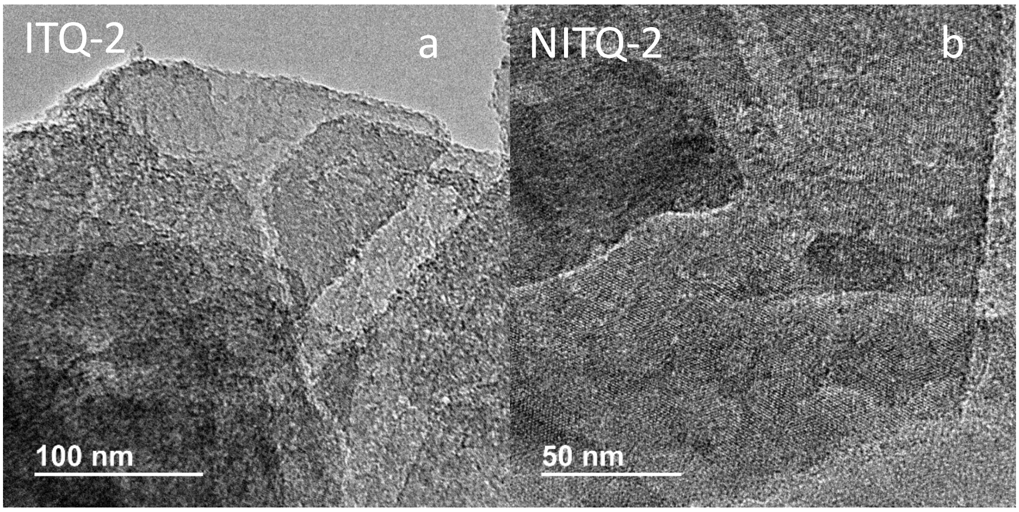

Figure S3. TEM images of ITQ-2 (a) and NITQ-2 (b)

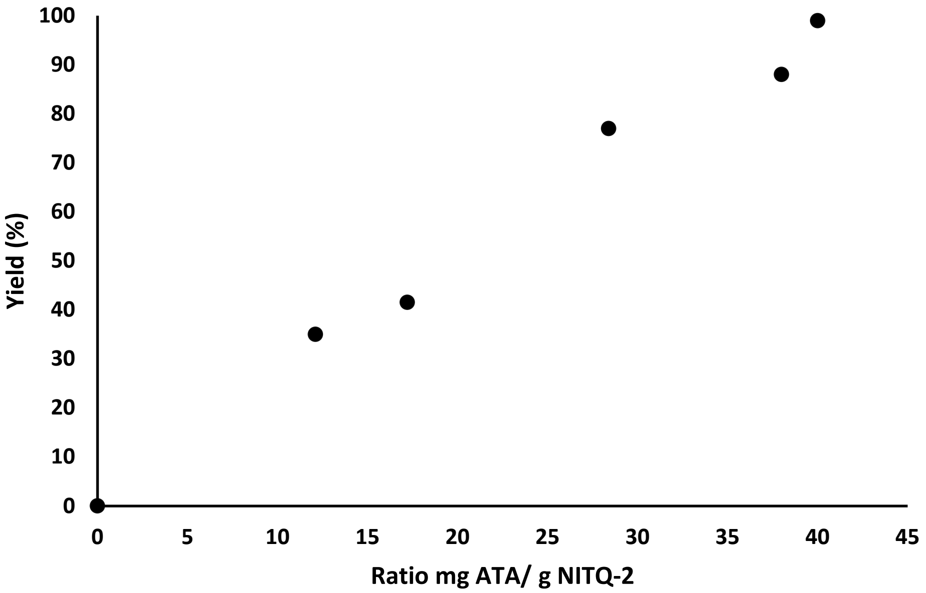

Figure S4. Influence of the enzyme loading on the biocatalyst activity

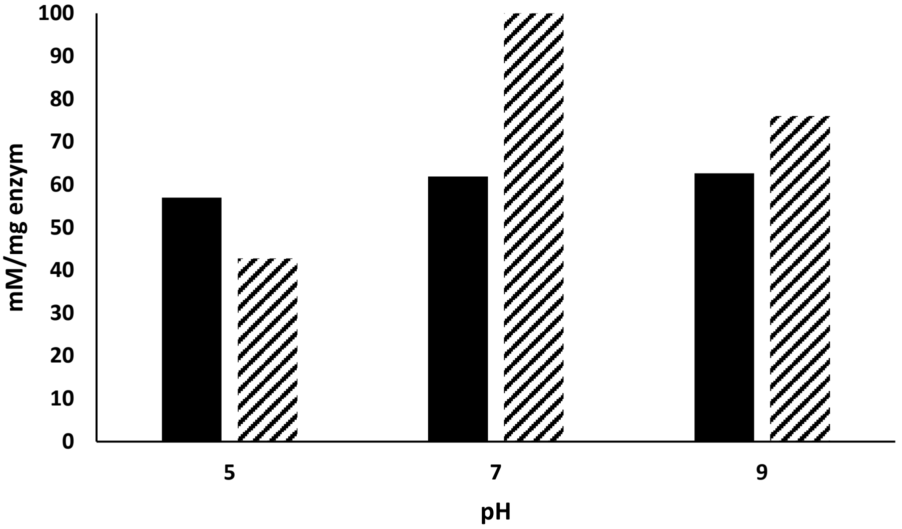

Figure S5. Relative activity as a function of pH for free and immobilized transaminase (Black) ATA(S)@NITQ-2 and (lined) free ATA(S).

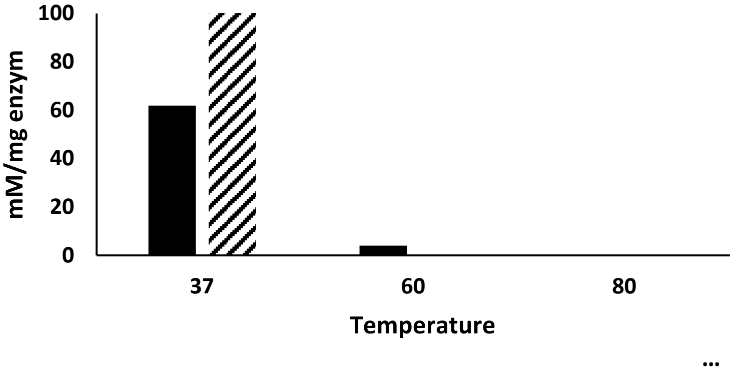

Figure S6. Relative activity as a function of temperature for free and immobilized transaminase (Black) ATA(S)@NITQ-2 and (lined) free ATA(S)

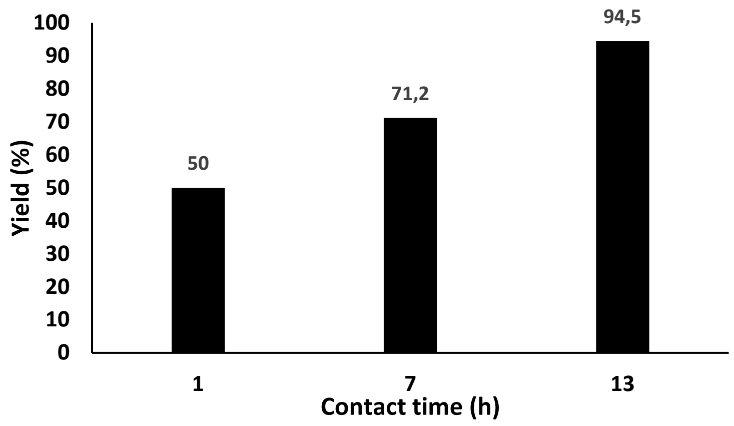

Figure S7. Evaluation of the contact time in flow reactor for the transamination of 4-(4-methoxyphenyl)-2-butanone to (S)-4-(4-methoxyphenyl)-2-butanamine. Reaction conditions: ATA(S)@NITQ-2 (208 mg), (molar ratio substrate/cofactor = 15), 22 mL of solvent isopropylamine/phosphate buffer pH 7, 100 mM (50/50 v/v) and DMSO (10 %) as co-solvent, flow 0.25-3 mL/h at 37 °C.

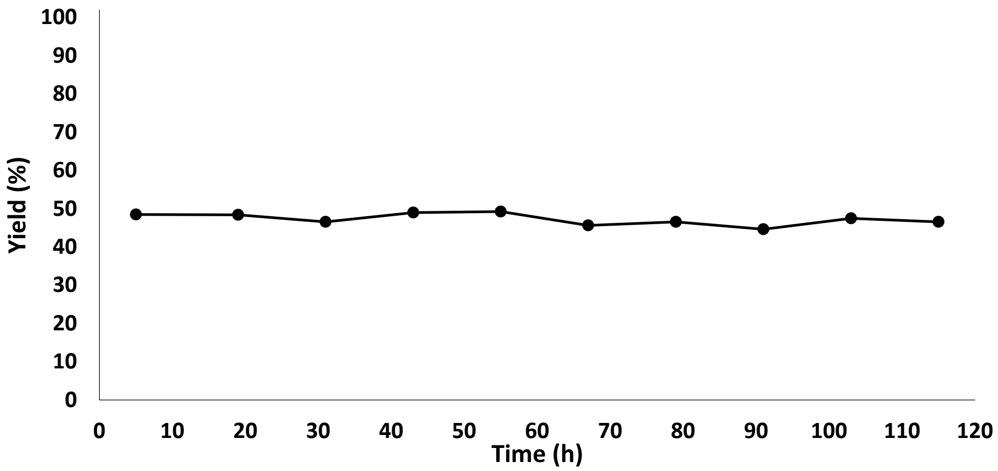

Figure S8. Study of the stability of ATA(S)@NITQ-2 in the transamination of 4-(4-methoxyphenyl)-2-butanone to (S)-4-(4-methoxyphenyl-2-butananime in a continuous reactor. Reaction conditions: ATA(S)@NITQ-2 (208 mg), 4-(4-methoxyphenyl)-2-butanone (15 mmol L^{-1}), PLP (1 mmol L^{-1}), solvent: isopropylamine/phosphate buffer pH 7, 100 mM (50/50 v/v) and DMSO (10 %) as co-solvent, flow 3 mL/h at 37 °C.

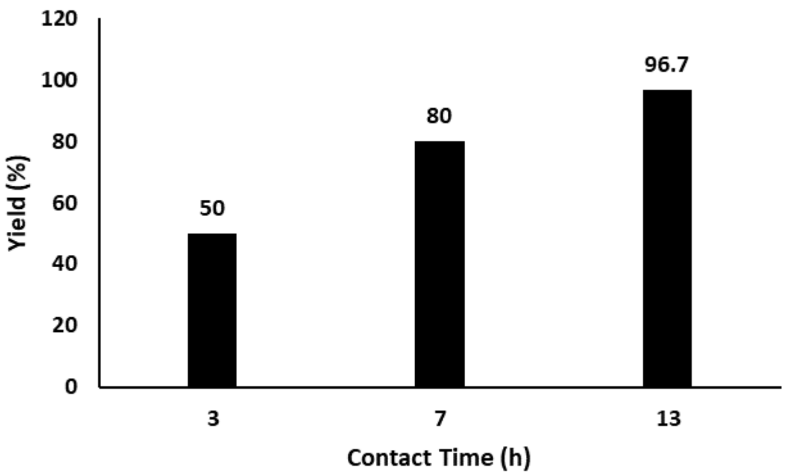

Figure S9. Evaluation of the contact time in flow reactor for the transamination of 4-phenyl-2-butanone to (S)-4-phenyl-2-butananime. Reaction conditions: ATA(S)@NITQ-2 (208 mg), (molar ratio substrate/cofactor = 15), 22 mL of solvent isopropylamine/phosphate buffer pH 7, 100 mM (50/50 v/v) and DMSO (10 %) as cosolvent, flow 0.25-1 mL/h at 37 °C.

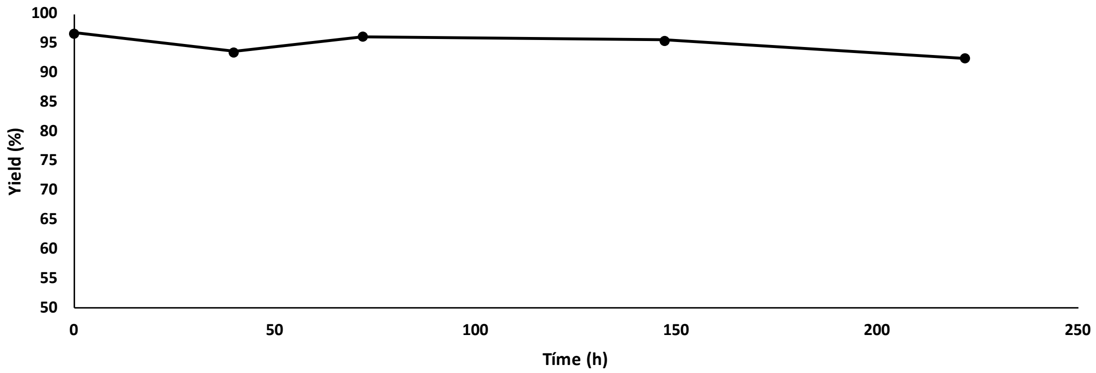

Fig S10. Results of the transamination of 4-phenyl-2-butanone to (S)-4-phenyl-2-butananime in a continuous reactor using ATA(S)@NITQ-2 as the biocatalyst. Reaction conditions: ATA(S)@NITQ-2 (208 mg), 4-phenyl-2-butanone (15 mmol L^{-1}), PLP (1 mmol L^{-1}), solvent: isopropylamine/phosphate buffer pH 7, 100 mM (50/50 v/v) and DMSO (10 %) as co-solvent, flow 0.25 mL/h at 37 °C.

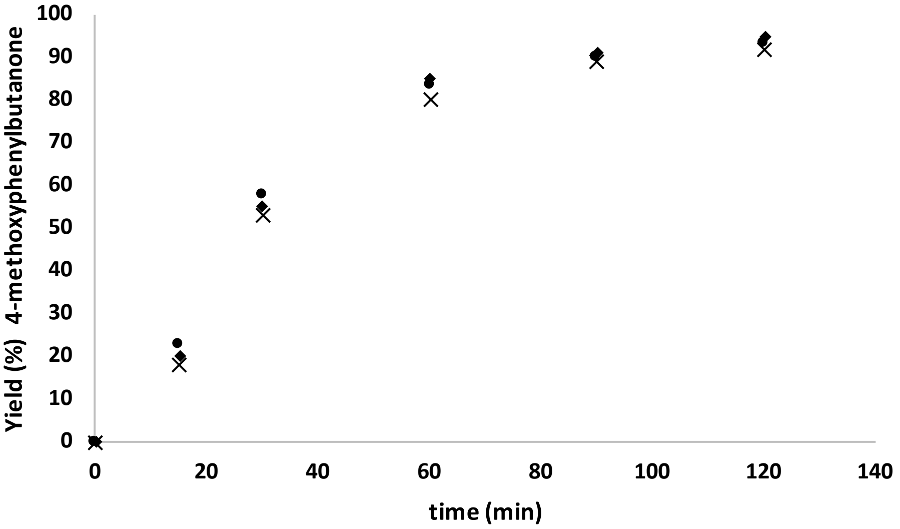

Figure S11. Reusability of the one-pot reaction of 4-methoxybenzaldehyde and acetone in a SCBR reactor using Pd/MgO as catalyst. Reaction conditions: 4-methoxybenzaldehyde (1.94 mmol), acetone (70 mmol), Pd/MgO (5 wt%), 5 bar (H_2), 120ºC, 2h. Reaction cycle 1(•), cycle 2 (×), cycle 3 (♦).

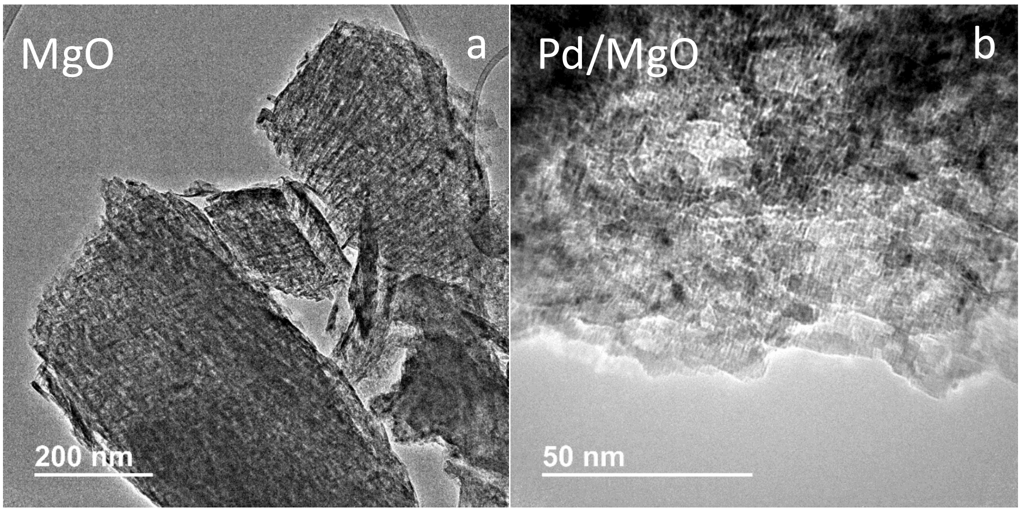

Figure S12. TEM images of MgO (a) and Pd/MgO (b).

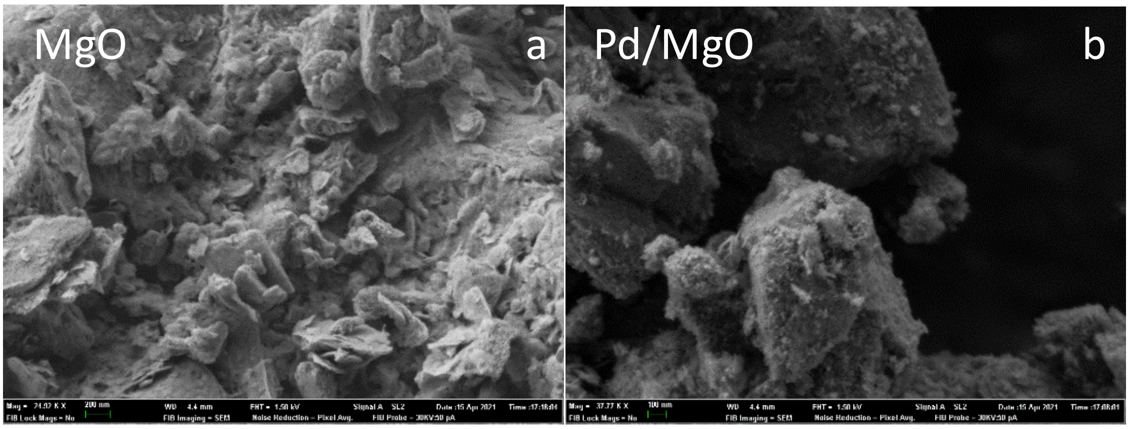

Figure S13. SEM images MgO (a) and Pd/MgO (b).

Table S1. Elemental analysis of the zeolite ITQ-2 and NITQ-2.

Material	N%	C%	H%
ITQ-2	-	0.366	0.296
NITQ-2	1.774	9.089	1.798

Table S2. BET analysis of the support.

Material	BET ($m^2 \cdot g^{-1}$)
ITQ-2	592
NITQ-2	375

Table S3. Evaluation of the contact time in flow reactor for the transamination of 4-methoxyphenyl-2-butanone to 4-methoxyphenyl-(S)-2-butanamine.

Flow (mL/h)	CT (h)	% Conversion
0.25	13	94.5
0.5	7	71.2
3	1	50

Table S4. Results of yield, productivity and operational stability of different supported transaminases for the amination of different carbonyl compounds reported in literature.

Article	Enzyme	Carrier used for Immobilization (strategy)	Reactor	Yield (%)	Productivity µmol/mgBiocat (µmol/mgEnzyme)	Operational stability (time)
[7]	Transaminase from Vibrio fluvialis	Modified cellulose (Covalent interaction)	Batch<br>Cont.	31<br>80	0.155(3.68)<br>0.078(3.63)	48h<br>180min
[8]	ATA-47<br>c-Lecta	Relizyme HA403 (Electrostatic interaction)	Batch	75<br>75	3.27(36)<br>1.63(18)	(250h) ATA-S<br>(250h) ATA-R
[9]	Transaminase Chromobacterium violaceum	Resin EziG (His6-tagged interaction)	Batch	42	3.15(nd)	24h
[10]	Transaminase Cromobacterium violaceum	Bisepoxide-activated polymeric resins (Covalent interaction)	Batch<br>Cont.	49<br>50	nd(93)<br>nd(114)	19h<br>8h
[11]	Transaminase wild type from C-Lecta	PVA gel Lentikats (entrapment)	Cont.	90	0.199(3.99)	21days
[12]	ATA-W12	AminoEthylenediamine-Modified Epoxide Supports (Covalent interaction)	Batch<br>Cont.	96<br>95	0.115(nd)<br>4.37(nd)	24h<br>24h
[13]	Transaminase from a Burkholderia sp.	Epoxy resin carrier (Covalent interaction)	Batch<br>Cont.	90<br>99	nd(3.35)<br>nd(61.91)	24h<br>38h
[14]	ω-transaminase from Halomonas elongata	Polymethacrylate-based porous bead carrier (Covalent interaction)	Batch<br>Cont.	70<br>84	0.102(1.36)<br>0.0256(0.341)	9h<br>240min
[15]	D-amino acid transaminase (DAT)	UiO-66-NH2 (Electrostatic interacion)	Batch<br>Batch (big scale)	77<br>70	3.56(nd)<br>0.7(nd)	-<br>12h
[16]	ATA-025	Epoxyacrylate resin (covalently)	Batch<br>Cont.	99<br>99	1.3(13)<br>1.3(16.25)	25h<br>20h
[17]	Transaminase from Vibrio fluvialis	Resin EziG (His6-tagged interaction)	Cont.	99	0.3(3)	4h
[18]	Bacillus megaterium	UiO-66-N	Batch (big scale)	80.5	nd (4.02)	24h
This work	ATA-47	NITQ2 zeolite (Electrostatic interaction)	Cont.	93	8.09(210)	22days

References:

1. Koszelewski D, Müller N, Schrittwieser JH *et al.* Immobilization of ω-transaminases by encapsulation in a sol-gel/celite matrix. *Journal of Molecular Catalysis B: Enzymatic* 2010;**63**:39–44.

2. Corma A, Fornes V, Pergher SB *et al.* Delaminated zeolite precursors as selective acidic catalysts. *Nature* 1998;**396**:353–6.

3. Corma A, Fornes V, Rey F. Delaminated zeolites: An efficient support for enzymes. *Advanced Materials* 2002;**14**:71–4.

4. Corma A, Fornés V, Jordá JL *et al.* Electrostatic and covalent immobilisation of enzymes on ITQ-6 delaminated zeolitic materials. *Chemical Communications* 2001;**5**:419–20.

5. Smith PK, Krohn RI, Hermanson GT *et al.* Measurement of protein using bicinchoninic acid. *Analytical Biochemistry* 1985;**150**:76–85.

6. Sheldon RA, van Pelt S. Enzyme immobilisation in biocatalysis: Why, what and how. *Chemical Society Reviews* 2013;**42**:6223–35.

7. de Souza SP, Junior II, Silva GMA *et al.* Cellulose as an efficient matrix for lipase and transaminase immobilization. *RSC Adv* 2016;**6**:6665–71.

8. Neto W, Schürmann M, Panella L *et al.* Immobilisation of ω-transaminase for industrial application: Screening and characterisation of commercial ready to use enzyme carriers. *Journal of Molecular Catalysis B: Enzymatic* 2015;**117**:54–61.

9. Engelmark Cassimjee K, Kadow M, Wikmark Y *et al.* A general protein purification and immobilization method on controlled porosity glass: biocatalytic applications. *Chemical Communications* 2014;**50**:9134.

10. Abaházi E, Sátorhelyi P, Erdélyi B *et al.* Covalently immobilized Trp60Cys mutant of w-transaminase from Chromobacterium violaceum for kinetic resolution of racemic amines in batch and continuous-flow modes. *Biochemical Engineering Journal* 2018;**132**:270–8.

11. Bajić M, Plazl I, Stloukal R *et al.* Development of a miniaturized packed bed reactor with ω-transaminase immobilized in LentiKats®. *Process Biochemistry* 2017;**52**:63–72.

12. Wang X, Xie Y, Wang Z *et al.* Efficient Synthesis of (S)-1-Boc-3-aminopiperidine in a Continuous Flow System Using ω-Transaminase-Immobilized Amino-Ethylenediamine-Modified Epoxide Supports. *Organic Process Research & Development* 2022;**26**:351–1359

13. Zhang XJ, Fan HH, Liu N *et al.* A novel self-sufficient biocatalyst based on transaminase and pyridoxal 5′-phosphate covalent co-immobilization and its application in continuous biosynthesis of sitagliptin. *Enzyme and Microbial Technology* 2019;**130**:109362.

14. Romero-Fernandez M, Paradisi F. Biocatalytic access to betazole using a one-pot multienzymatic system in continuous flow. *Green Chemistry* 2021;**23**:4594–603.

15. Wang B, Zhou J, Zhang XY *et al.* Covalently immobilize crude D-amino acid transaminase onto UiO-66-NH_2 surface for D-Ala biosynthesis. *International Journal of Biological Macromolecules* 2021;**175**:451–8.

16. Petri A, Colonna V, Piccolo O. Asymmetric synthesis of a high added value chiral amine using immobilized ω-transaminases. *Beilstein Journal of Organic Chemistry* 2019;**15**:60–6.

17. Mattey AP, Ford GJ, Citoler J *et al.* Development of Continuous Flow Systems to Access Secondary Amines Through Previously Incompatible Biocatalytic Cascades**. *Angewandte Chemie - International Edition* 2021;**60**:18660–5.

18. Yu J, Zong W, Ding Y *et al.* Fabrication of ω-Transaminase@Metal-Organic Framework Biocomposites for Efficiently Synthesizing Benzylamines and Pyridylmethylamines. *Advanced Synthesis and Catalysis* 2022;**364**:380–90.
